# VCAM-1–targeted MRI Improves Detection of the Tumor-brain Interface

**DOI:** 10.1158/1078-0432.CCR-21-4011

**Published:** 2022-03-01

**Authors:** Vinton W.T. Cheng, Nicholas de Pennington, Rasheed Zakaria, James R. Larkin, Sébastien Serres, Manjima Sarkar, Matthew A. Kirkman, Claire Bristow, Paula Croal, Puneet Plaha, Leticia Campo, Michael A. Chappell, Simon Lord, Michael D. Jenkinson, Mark R. Middleton, Nicola R. Sibson

**Affiliations:** 1 Department of Oncology, University of Oxford, Oxford, United Kingdom.; 2 Leeds Institute of Medical Research, University of Leeds, Leeds, United Kingdom.; 3 Department of Neurosurgery, The Walton Centre NHS Foundation Trust, Liverpool, United Kingdom.; 4 Faculty of Health and Life Sciences, University of Liverpool, Liverpool, United Kingdom.; 5 School of Life Sciences, University of Nottingham, Nottingham, United Kingdom.; 6 UCL Institute for Education, University College London, London, United Kingdom.; 7 Mental Health and Clinical Neurosciences & Sir Peter Mansfield Imaging Centre, School of Medicine, University of Nottingham, Nottingham, United Kingdom.; 8 Nottingham Biomedical Research Centre, Queens Medical Centre, University of Nottingham, Nottingham, United Kingdom.; 9 Nuffield Department of Surgery, University of Oxford and Department of Neurosurgery, Oxford University Hospitals NHS Trust, Oxford, United Kingdom.; 10 Institute of Systems, Molecular and Integrative Biology, University of Liverpool, Liverpool, United Kingdom.; 11 Experimental Cancer Medicine Centre, Department of Oncology, University of Oxford, Oxford, United Kingdom.; 12 Oxford National Institute for Health Research Comprehensive Biomedical Research Centre, Oxford, United Kingdom.

## Abstract

**Purpose:**

Despite optimal local therapy, tumor cell invasion into normal brain parenchyma frequently results in recurrence in patients with solid tumors. The aim of this study was to determine whether microvascular inflammation can be targeted to better delineate the tumor-brain interface through vascular cell adhesion molecule-1 (VCAM-1)-targeted MRI.

**Experimental Design:**

Intracerebral xenograft rat models of MDA231Br-GFP (breast cancer) brain metastasis and U87MG (glioblastoma) were used to histologically examine the tumor-brain interface and to test the efficacy of VCAM-1–targeted MRI in detecting this region. Human biopsy samples of the brain metastasis and glioblastoma margins were examined for endothelial VCAM-1 expression.

**Results:**

The interface between tumor and surrounding normal brain tissue exhibited elevated endothelial VCAM-1 expression and increased microvessel density. Tumor proliferation and stemness markers were also significantly upregulated at the tumor rim in the brain metastasis model. *T*_2_*-weighted MRI, following intravenous administration of VCAM-MPIO, highlighted the tumor-brain interface of both tumor models more extensively than gadolinium-DTPA–enhanced *T*_1_-weighted MRI. Sites of VCAM-MPIO binding, evident as hypointense signals on MR images, correlated spatially with endothelial VCAM-1 upregulation and bound VCAM-MPIO beads detected histologically. These findings were further validated in an orthotopic medulloblastoma model. Finally, the tumor-brain interface in human brain metastasis and glioblastoma samples was similarly characterized by microvascular inflammation, extending beyond the region detectable using conventional MRI.

**Conclusions:**

This work illustrates the potential of VCAM-1–targeted MRI for improved delineation of the tumor-brain interface in both primary and secondary brain tumors.

**Translational Relevance**

Current clinical neuroimaging techniques for characterizing brain tumors are limited in their capacity to detect the invasive margin. Failure to detect and adequately treat the invasive tumor margin contributes to future recurrence and increased patient mortality. We present a novel application of a molecularly targeted MRI contrast agent against VCAM-1, a sensitive marker for microvascular inflammation, to better delineate the interface between tumor and adjacent brain tissue compared with conventional MRI techniques. Thus, VCAM-1–targeted MRI presents an opportunity to augment existing imaging paradigms to tailor treatment planning and delivery. Moreover, avoiding inadvertent treatment of uninvolved tissue would reduce the risk of side effects and therapy-related morbidities.

## Introduction

Local brain invasion is a major problem in the oncological management of brain tumors owing to risk of damaging eloquent brain in the course of treatment, either by surgery or radiation. Preserving neurologic function versus maximizing local tumor control is an ongoing predicament facing surgeons and radiation oncologists. The so-called “onco-functional balance” has been applied in the context of low-grade gliomas (1), but is similarly relevant in other invasive brain tumors. Glioblastoma, the most aggressive form of primary brain tumor, is characterized by diffuse infiltration of tumor cells into the surrounding brain parenchyma and this defining feature is a major contributor to poor survival outcomes. Recent studies also point to the infiltrative nature of brain metastases (2–4), albeit in a more heterogeneous fashion than in glioblastoma; thus, highlighting the need for better clinical detection of the tumor-brain interface.

Surgery and brain-directed radiotherapy are the main treatment modalities for both primary and metastatic brain tumors. Treatment planning in both settings is dependent on noninvasive imaging, particularly MRI. However, the limitations of current MRI techniques in accurately delineating the tumor, particularly the tumor-brain interface, are well documented (5–7). For brain metastases, despite optimal surgical treatment, local relapse occurs in a large proportion of patients with a recurrence rate at the original metastatic site, following gross total resection, of 46% (8). Meanwhile, at 12 months following standalone stereotactic radiosurgery treatment for a brain metastasis, 27.5% of patients experience recurrence within or adjacent to the treatment field (9). In the context of glioblastoma, despite combination therapy with both gross tumor resection and adjuvant focal radiotherapy, half of all patients treated would be expected to relapse within 5 months (10). These high treatment failure rates most likely reflect the presence of subclinical disease and/or treatment resistant clones within the adjacent, suboptimally treated brain parenchyma.

Advancing the existing management of brain tumors will require enhanced imaging techniques for better delineating the tumor-brain interface. In preclinical studies, we have shown that it is possible to augment the sensitivity of MRI for detecting subclinical micrometastases in the brain using a contrast agent based on microparticles of iron oxide (MPIO) targeted against the cell adhesion molecule, vascular cell adhesion molecule-1 (VCAM-1; refs. 11, 12). These data suggest that an invasive, proliferative tumor profile is associated with endothelial VCAM-1 upregulation.

On this basis, we hypothesized that application of VCAM-1–targeted MRI may enable more sensitive delineation of the tumor-brain interface. Our initial aim, therefore, was to determine whether the tumor-brain interface in rat models of brain metastasis and glioblastoma show upregulation of VCAM-1. Next, we wanted to assess the sensitivity of VCAM-1–targeted MPIO (VCAM-MPIO) in conjunction with *T*_2_*-weighted MRI for detection of the tumor-brain interface compared with conventional MRI, and further extend this analysis to a xenograft model of medulloblastoma to assess its broader applicability. Finally, we aimed to evaluate the relationship between VCAM-1 upregulation, the tumor margin, and conventional MRI indices in human brain metastasis and glioblastoma samples.

## Materials and Methods

### Cell lines

Three human-derived cell lines were used: MDA231Br-GFP cells (subclone of metastatic breast carcinoma that preferentially metastasizes to the brain; kind gift from Prof. P. Steeg), U87MG cells (glioblastoma; kind gift from Prof. A. Harris) and DAOY cells (medulloblastoma; kind gift from Dr. Maike Glitsch). Following resuscitation from liquid nitrogen storage, no cell line underwent more than five passages prior to *in vivo* injection and were cultured in DMEM (Sigma-Aldrich) supplemented with 10% FCS (Thermo Fisher Scientific) and 1% l-glutamine (Life Technologies) in a humidified incubator at 37°C with 5% CO_2_. All cell lines underwent routine *Mycoplasma* testing (MycoAlert *Mycoplasma* Detection Kit, Lonza). Short tandem repeat profiling has not been performed; however, work was conducted from the original cell stock for all cell lines. Moreover, all experiments within a study were run from the same passage from this original stock to ensure reproducibility, as far as is possible.

### Experimental models

Female RNU nude rats (5–6 weeks old; 200 ± 20 g; Charles River Laboratories) were anesthetized with 2%–3% isoflurane in oxygen and injected in the left striatum, with 5 × 10^3^ MDA231Br-GFP or U87MG cells in 0.5 μL PBS, as described previously (13). For the medulloblastoma model, 10^4^ DAOY cells in 1 μL PBS were injected intracerebrally in the cerebellar vermis, as described previously (14). Animals underwent MRI at day 28 after intrastriatal injection for the MDA231Br-GFP and U87MG models, and at week 15 for the DAOY model. All animal experiments were approved by the University of Oxford Clinical Medicine Ethics Review Committee and the UK Home Office [Animals (Scientific Procedures) Act 1986], and conducted in accordance with the University of Oxford Policy on the Use of Animals in Scientific Research, the ARRIVE Guidelines and Guidelines for the Welfare and Use of Animals in Cancer Research (15).

### IHC of rat brain tissue

Brain tissue sections from tumor-bearing mice and control cohorts were immunostained for VCAM-1 (μg/mL catalog no. 14-1060-85; eBioscience), CD31 (2 μg/mL catalog no. AF3628; R&D Systems), Ki67 (1:100 catalog no. ab16667; Abcam), SOX2 (1:100 catalog no. SAB5500176; Sigma-Aldrich), and nestin (1:100 catalog no. ab105389; Abcam) expression. Tumor cells were identified with anti-vimentin (2.5 μg/mL catalog no. ab92547; Abcam) staining. Stained slides were digitized via an Aperio CS2 digital pathology slide scanner (Leica) and the images were analyzed with Aperio ImageScope (v. 12.3.3; Leica). For detailed information on histologic analysis, see Supplementary Materials and Methods.

### Rat brain MRI

MRI data were acquired using a 9.4T MRI spectrometer. On the day of imaging, tumor-injected rats were anesthetized with 2%–3% isoflurane in 70% nitrogen: 30% oxygen and injected intravenously via a tail vein with 4 mg Fe/kg body mass VCAM-MPIO (*n* = 5–8 per group) or IgG-MPIO (*n* = 6 per group) in 100 µL saline. A further cohort of control nude rats, with intrastriatal injection of PBS only, had VCAM-MPIO or IgG-MPIO intravenously administered as above (*n* = 3 per group). At 30 minutes after MPIO injection, animals were positioned in a customized cradle inside a quadrature birdcage coil (72 mm internal diameter; RAPID Biomedical GmbH). Respiration was monitored, and body temperature maintained at approximately 37°C. For detailed information on the antibody-MPIO synthesis, MRI sequences employed, image processing, and co-registration analysis, see Supplementary Materials and Methods.

### Human MRI and brain metastasis biopsy acquisition

Human brain metastasis samples were obtained at The Walton Centre NHS Foundation Trust by image-guided biopsy (ethics reference: 11/WNo03/02), as described previously (16, 17). Briefly, patients underwent MRI prior to surgery, with pre- and post-gadolinium *T*_1_-weighted and diffusion-weighted sequences. The diffusion-weighted images were converted into apparent diffusion coefficient (ADC) maps, which were then overlaid onto the structural post-gadolinium images, to identify the tumor and specifically its “leading edge” at the tumor-brain interface. Guided by these fused sequences, samples were taken using a Suretrak probe mounted on biopsy forceps. Radiologically assessed tumor volumes and tumor biopsy characteristics according to histology are reported in Supplementary Tables S1 and S2, respectively.

Three cases of human brain metastasis, each with three to four biopsy locations, were obtained for breast cancer (*n* = 10 samples), lung adenocarcinoma (*n* = 11 samples), and melanoma (*n* = 12 samples); samples were selected to contain tissue at the tumor-brain interface. The corresponding MR images for each patient were anonymized and downloaded as DICOM files for subsequent offline analysis. Moreover, the vectors specifying the location of this sample (“edge”), along with one from the tumor interior (“core”) were extracted from the image-guidance software postoperatively (StealthStation S7, Medtronic Inc.). These allowed identification of specific regions of interest (ROI) that colocalized to the tissue samples taken intraoperatively (see example in Supplementary Fig. S1). For detailed information on the MRI sequences and analysis, see Supplementary Materials and Methods.

### Human MRI and glioblastoma biopsy acquisition

Human glioblastoma samples were obtained at the Oxford University Hospitals NHS Foundation Trust by image-guided biopsy, as part of the IMAGO study (ISRCTN86522205). Briefly, previously untreated patients with glioblastoma scheduled for resection or debulking were consented to undergo additional perioperative MRI and navigated surgical biopsies. For the purposes of the current study, only the standard anatomic MRI (post-gadolinium *T*_1_-weighted and diffusion-weighted) sequences and biopsies conforming to sites clinically determined to be at the tumor-brain interface were utilized.

Seven cases of human glioblastoma, each with between one to two navigated biopsy locations, were obtained (*n* = 9 samples). The corresponding MR images for each patient were anonymized and downloaded as DICOM files for subsequent offline analysis. Analysis followed the same pattern as outlined for the human brain metastasis samples. Additional detail of MRI sequences used is outlined in the Supplementary Materials and Methods.

### IHC of human brain tissue

Human brain metastasis and glioblastoma biopsies were processed into formalin-fixed and paraffin embedded specimens and sectioned at 6 μm. Tissue sections were deparaffinized and rehydrated, then immunohistochemically stained for VCAM-1 (20 μg/100 uL catalog no. sc8304; Santa Cruz Biotechnology). Adjacent sections were stained for tumor-specific markers: anti-cytokeratin purified clone CAM5.2 (12.5 μg/mL catalog no. 345779; BD Biosciences) for breast cancer and lung adenocarcinoma, and anti-melan A (1:100 catalog no. ab5106; Abcam) for melanoma. Additional sections were stained with anti-CD34 antibody (1:50 catalog no. M7165; Dako) for vascular characterization. In the case of the glioblastoma specimens, insufficient tissue was available for tumor-specific or blood vessel staining. For detailed information on histologic analysis, see Supplementary Materials and Methods.

### Statistical analysis

Differences in vimentin and CD31 expression were assessed by repeated measures one-way ANOVA tests, with *post hoc* Bonferroni multiple comparison tests used to identify specific differences between groups. As the data for VCAM-1 expression were not normally distributed, nonparametric analysis was employed by Friedman test, with *post hoc* Dunn tests for detection of between-group differences. Tumor cell Ki67, nestin, and SOX2 expression were normalized to tumor cell density and differences in expression were examined by matched Wilcoxon tests. For preclinical MRI hypointensity volume, differences between animal cohorts for both tumor types were identified by two-way ANOVA, with *post hoc* Tukey tests employed for between-group differences. The difference in extension of hypointense signal on *T*_2_*-weighted MRI beyond *T*_1_-weighted gadolinium contrast enhancement was assessed by Student *t* test.

For human MRI, differences in normalized grey pixel intensity or ADC values were assessed by repeated measures one-way ANOVA. *Post hoc* Tukey multiple comparisons tests were used to detect individual between-group differences. Microvessel density across the peritumoral brain parenchyma was assessed by one-way ANOVA and *post hoc* Tukey tests. The relationship between VCAM-expressing endothelium and distance from the tumor edge was assessed by Pearson product moment correlation.

All statistical analyses were two-sided, reported at a significance level of 0.05 and performed in GraphPad Prism (v.9; GraphPad Software).

### Data availability

The data generated in this study are available within the article and its Supplementary Data.

## Results

### The tumor-brain interface in rat models display distinct biological features from the tumor core

**Figure 1.**
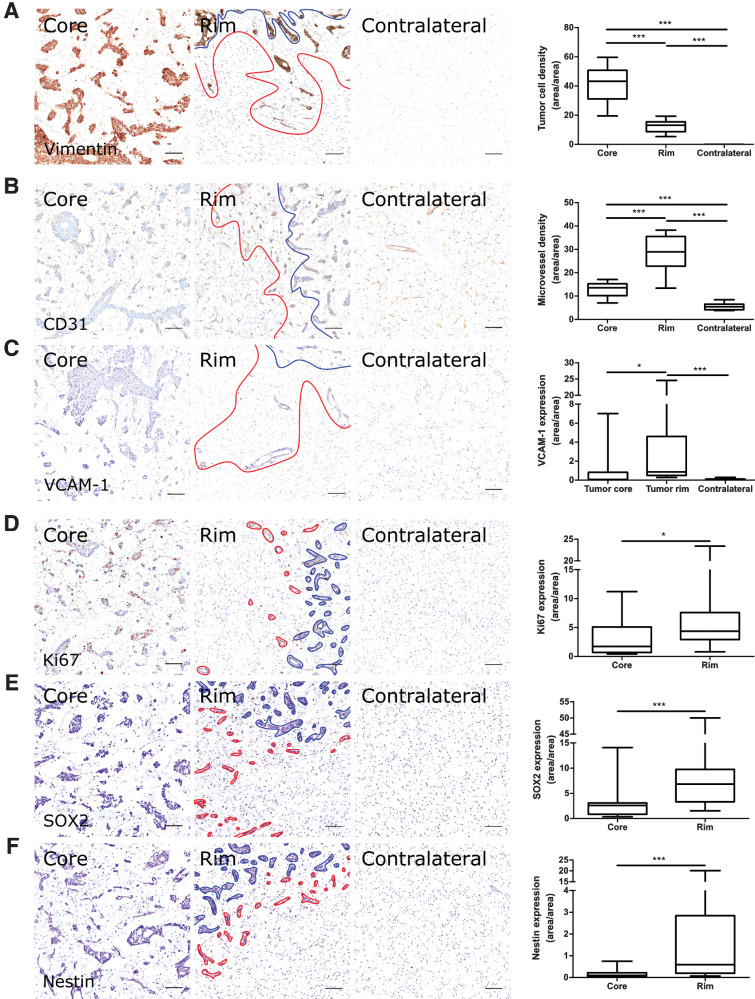
Microvascular and tumor cell biology at the tumor rim in MDA231Br-GFP brain metastases. Representative histologic sections of rat brains, intrastriatally injected with MDA231Br-GFP tumor cells, from the tumor core, tumor rim, and the contralateral striatum, with corresponding box and whisker plots of marker expression in each region. Data shown as median ± interquartile range. Sections were immunohistochemically stained (brown) for tumor cell marker vimentin (**A**), endothelial marker CD31 (**B**), cell adhesion molecule VCAM-1 (**C**), cell proliferation marker Ki67 (**D**), and two stemness markers: SOX2 (**E**) and nestin (**F**). Scale bar = 100 μm. *, *P* < 0.05; ***, *P* < 0.001; *n* = 8; *post hoc* Bonferroni multiple comparison test for tumor cell density and microvessel density, and *post hoc* Dunn test for VCAM-1 expression. Tumor core was delineated from the infiltrative border as indicated by the blue and red lines, respectively. Expression of Ki67, nestin and SOX2 has been normalized to tumor area. *, *P* < 0.05; ***, *P* < 0.001; matched Wilcoxon test.

**Figure 2.**
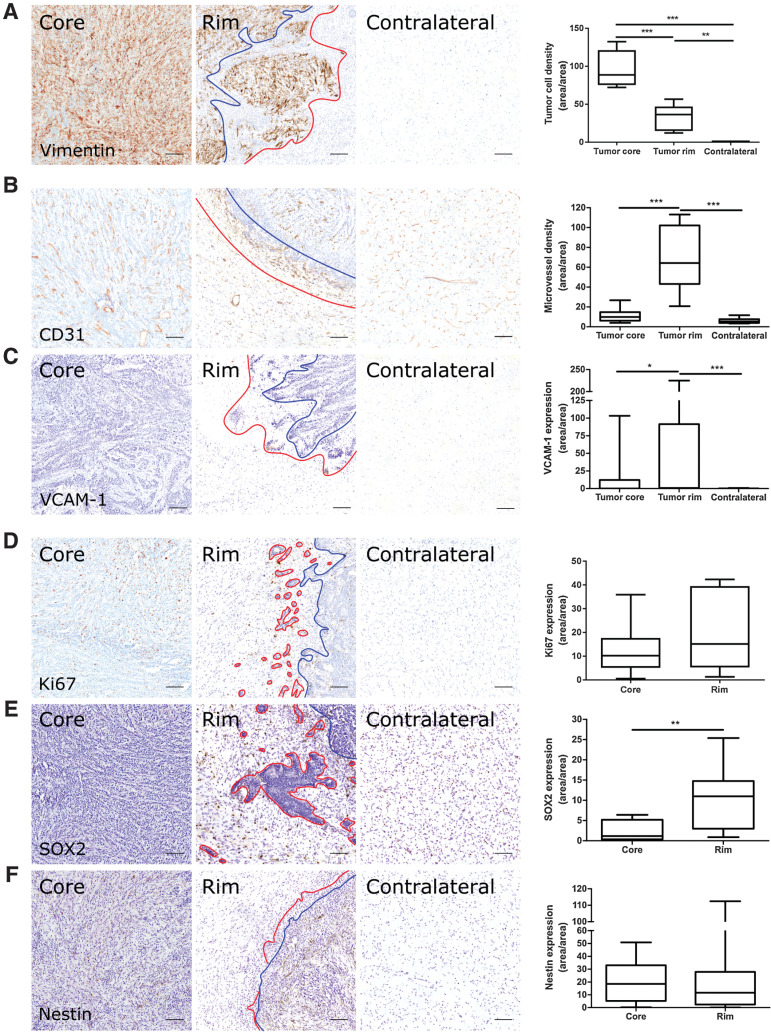
Microvascular and tumor cell biology at the tumor rim in U87MG glioblastoma. Representative histologic sections of rat brains, intrastriatally injected with U87MG tumor cells, from the tumor core, tumor rim, and the contralateral striatum, with corresponding box and whisker plots of marker expression in each region. Data shown as median ± interquartile range. Sections were immunohistochemically stained (brown) for tumor cell marker vimentin (**A**), endothelial marker CD31 (**B**), cell adhesion molecule VCAM-1 (**C**), cell proliferation marker Ki67 (**D**), and two stemness markers: SOX2 (**E**) and nestin (**F**). Scale bar = 100 μm. *, *P* < 0.05; **, *P* < 0.01; ***, *P* < 0.001; *n* = 5; *post hoc* Bonferroni multiple comparison test for tumor cell density and microvessel density, and *post hoc* Dunn test for VCAM-1 expression. Tumor core was delineated from the infiltrative border as indicated by the blue and red lines, respectively. Expression of Ki67, nestin, and SOX2 has been normalized to tumor area. **, *P* < 0.01; matched Wilcoxon test.

In both the brain metastasis (MDA231Br-GFP) and glioblastoma (U87MG) models, all animals exhibited intrastriatal tumors of sufficient size to be detected on MRI (MDA231Br-GFP 13.8 ± 10.4 μL; U87MG 28.2 ± 16.2 μL, estimated from *T*_2_-weighted imaging), with areas of poorly demarcated border (Supplementary Fig. S2). Serial sections were used to spatially interrogate antigen expression of vimentin, VCAM-1, CD31, Ki67, nestin, and SOX2 in both tumor cells and the adjacent stroma, specifically at the tumor core, tumor rim, and contralateral striatum (Figs. 1 and 2). Tumor core was defined as the central mass comprising contiguous tumor cells and encompassing at least 90% of the tumor cross-sectional area. Meanwhile, the tumor rim was defined as the area containing isolated tumor cells, or clusters of tumor cells, at the periphery of the central mass that were detached from the tumor core or that disrupted the smoothness of the tumor boundary.

A significant increase in microvessel density was evident at the tumor-brain interface compared with both tumor core and contralateral striatum for both the MDA231Br-GFP and U87MG tumor models (repeated measures one-way ANOVA, *P* < 0.0001 for both models; Figs. 1B and 2B). Similarly, VCAM-1 expression was significantly greater in the tumor rim for both models (Friedman test, *P* < 0.001 for both models; Figs. 1C and 2C), with negligible VCAM-1 expression evident in the contralateral striatum. In both cases, *post hoc* pairwise statistical comparisons are shown in Figs. 1 and 2.

Tumor cell proliferation, when corrected for tumor cell density, showed a relative increase in Ki67 expression from the tumor core to the rim for MDA231Br-GFP (60.2%) and U87MG (32.4%) tumors, respectively; although this increase reached significance for the MDA231Br-GFP group (matched Wilcoxon test, *P* < 0.05; Fig. 1D) and not the U87MG group (Fig. 2D). Stemness markers, SOX2 and nestin, were differentially expressed in tumor cells at the invasive margin. SOX2 expression was significantly greater at the tumor rim than the core for both MDA231Br-GFP and U87MG models (matched Wilcoxon test, *P* < 0.001 and *P* < 0.01, respectively; Figs. 1E and 2E). Similarly, nestin expression was significantly greater at the tumor rim than the core in the MDA231Br-GFP tumors (matched Wilcoxon test, *P* < 0.001; Fig. 1F), although this was not replicated in the U87MG tumors (Fig. 2F).

### VCAM-MPIO MRI enables improved detection of the tumor-brain interface

**Figure 3.**
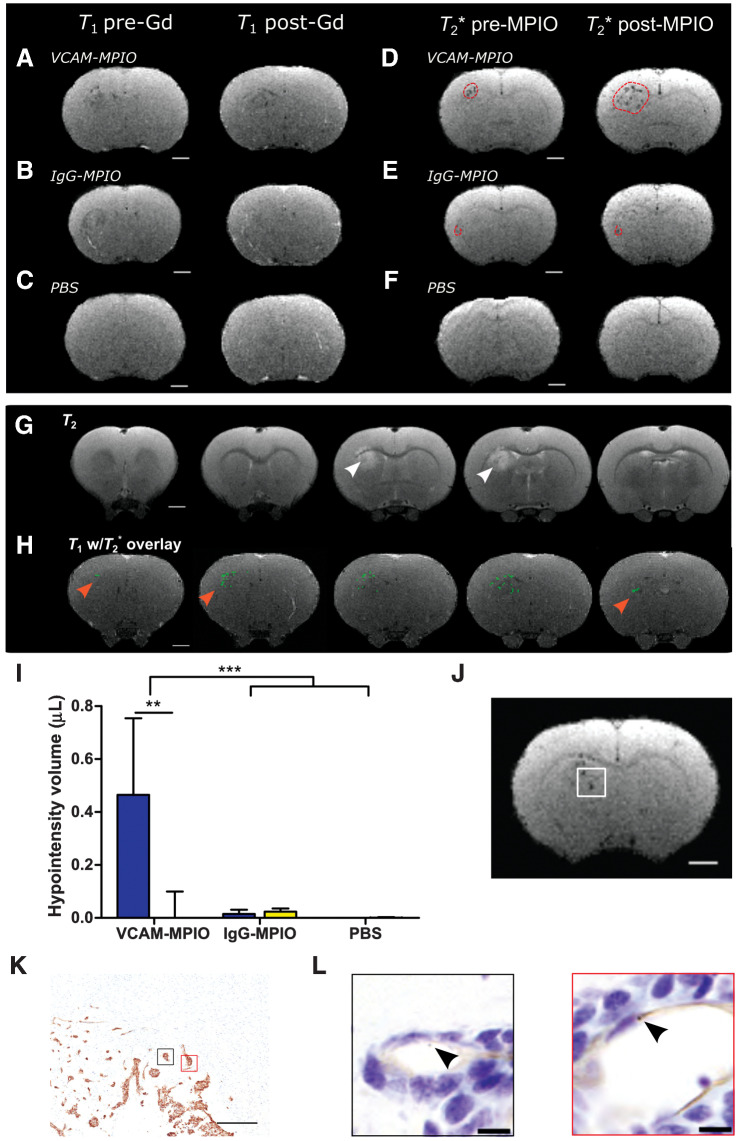
Anti-VCAM-1–targeted MRI reveals tumor margins in MDA231Br-GFP brain metastases. Pre-/post-gadolinium *T*_1_-weighted images from rat brains either with intrastriatal MDA231Br-GFP tumors (**A** and **B**) or injected intrastriatally with PBS (**C**). Matched pre-/post-MPIO *T*_2_*-weighted images from the same anatomic locations and same animals as for **A** and **B**, injected intravenously with either VCAM-MPIO contrast (**D**), or IgG-MPIO (**E**). **F,** Pre-/post-MPIO *T*_2_*-weighted images from a rat injected intrastriatally with PBS and VCAM-MPIO intravenously. Dashed red line encircles visible hypointense regions. **G,***T*_2_-weighted images showing areas of tumor-associated hyperintensity (white arrowhead). **H,** Representative MR images of MDA231Br-GFP tumor-bearing rat brain with hypointensities (green pixels) from *T*_2_*-weighted imaging overlaid on contrast-enhanced *T*_1_-weighted imaging. Note the green pixels extend into regions of the striatum with no associated *T*_2_ hyperintensity (red arrowheads). Scale bar = 2 mm. **I,** Graph showing volumes of hypointensities post-*T*_2_*-weighted MRI; data shown as mean ± SD, blue = ipsilateral striatum, yellow = contralateral striatum. **, *P* < 0.01; ***, *P* < 0.001; *post hoc* Tukey test (*n* = 8 MDA231Br-GFP groups and *n* = 3 PBS group). **J,** Representative *T*_2_*-weighted MR image slice of a MDA231Br-GFP tumor, with white box highlighting the invasive tumor margin confirmed histologically (**K**); scale bar = 500 μm. **L,** Corresponding high-power magnification of highlighted regions of interest (black and red boxes) from stained adjacent sections, demonstrating bound VCAM-MPIO beads (black arrowhead/brown spheres) in a VCAM-1–positive vessel lumen (brown); scale bar = 10 μm.

On the basis that all tumors showed upregulated VCAM-1 expression in the tumor periphery, associated with proliferative tumor cells and increased vascular density, we next assessed the sensitivity of VCAM-1–targeted MRI for detection of the tumor-brain interface as compared with the current clinical gold standard of gadolinium-enhanced *T*_1_-weighted imaging (Fig. 3). No MDA231Br-GFP tumors were visible on post-gadolinium *T*_1_-weighted images at 28 days posttumor cell injection (Fig. 3A and B), despite being relatively well established as determined histologically (Fig. 1A). No MRI-detectable changes were evident in control animals injected intrastriatally with PBS (Fig. 3C).

Prior to injection of MPIO contrast agents, a small number of hypointensities were visible in the ipsilateral striatum, corresponding to the location of the tumor, on *T*_2_*-weighted images (Fig. 3D and E). Following intravenous injection of VCAM-MPIO, however, MDA231Br-GFP tumor-bearing rats showed significantly increased volumes of hypointensities in the tumor-bearing striatum (Fig. 3D and G), consistent with binding of VCAM-MPIO to upregulated endothelial VCAM-1. Minimal hypointensities were seen in the contralateral striatum.

Because no post-gadolinium contrast changes were evident on *T*_1_-weighted images, VCAM-targeted MRI appears to be more sensitive than gadolinium enhancement to tumor presence, which was confirmed histologically (Supplementary Fig. S3). However, in the absence of gadolinium-induced contrast changes, *T*_2_-weighted imaging could be considered a clinical alternative, and this imaging modality further confirmed presence of well-established tumors (Fig. 3G; Supplementary Fig. S4). Nevertheless, it was also clear that the extent of hypointensities induced by the VCAM-MPIO on *T*_2_*-weighted images extended substantially beyond the area of hyperintensity on corresponding *T*_2_-weighted images (Fig. 3G and H). Although a single-imaging slice is shown for comparison in the above Figure, owing to differences in imaging resolution, multiple *T*_2_*-weighted image slices (thickness 120 μm) will, in fact, correspond to a single *T*_2_-weighted image slice (thickness 500 μm). In comparing stacked T_2_*-weighted images against the corresponding single *T_2_*-weighted slice, it can be seen more clearly that the hypointensities encompass the entire perimeter of the tumor (see example in Supplementary Fig. S5).

Neither control cohort, comprising tumor-bearing animals injected with the nonspecific anti-rat IgG-MPIO (Fig. 3E) or PBS-injected animals imaged with VCAM-MPIO (Fig. 3F), showed increased volumes of hypointensity in either the ipsilateral or contralateral striatum compared with precontrast agent injection (Fig. 3I). Moreover, histologic examination confirmed concordance with the MRI, as luminally bound VCAM-MPIO was localized to sites corresponding to the hypointense signal seen on *T*_2_*-weighted MRI (Fig. 3J–L).

**Figure 4.**
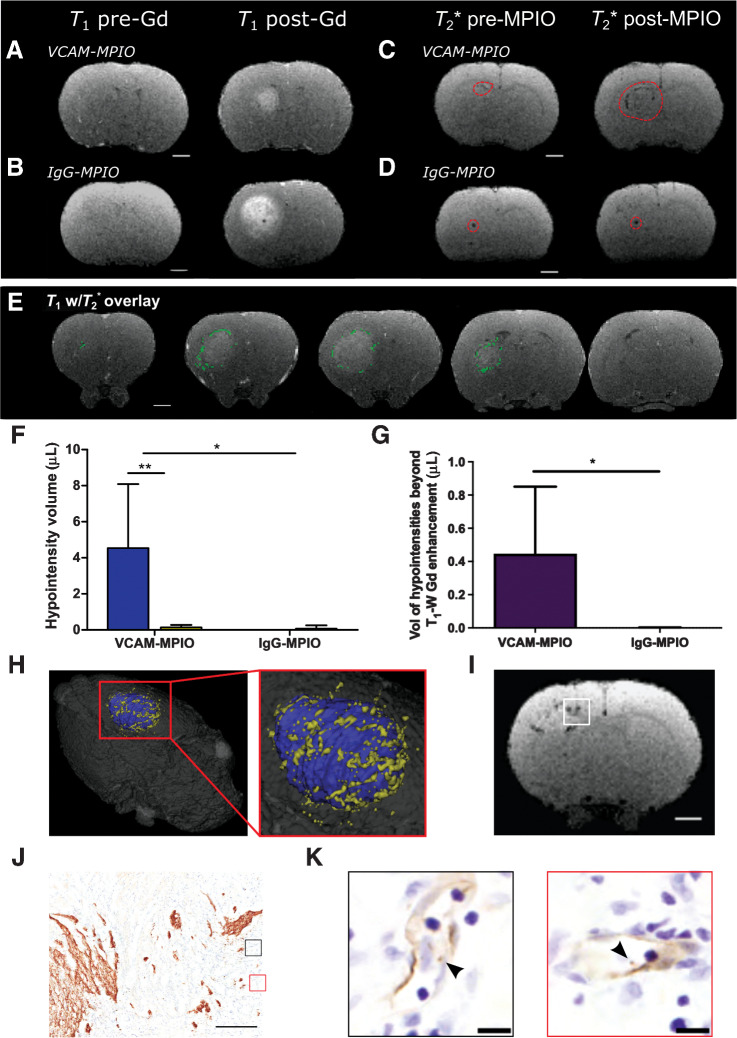
Anti-VCAM-1–targeted MRI reveals tumor margins in U87MG brain tumors. **A** and **B,** Pre-/post-gadolinium *T*_1_-weighted images from rat brains with intrastriatal U87MG tumors. Matched pre-/post-MPIO *T*_2_*-weighted images from rat brains with intrastriatal U87MG tumors, injected intravenously with VCAM-MPIO (**C**) or IgG-MPIO (**D**); dashed red line encircles visible hypointense regions. Scale bar = 2 mm. **E,** Representative images from U87MG tumor-bearing rat brain with hypointensities (green pixels) from *T*_2_*-weighted images overlaid on contrast-enhanced *T*_1_-weighted images. Scale bar = 2 mm. **F,** Graph showing volumes of hypointensities post-*T*_2_*-weighted MRI; data shown as mean ± SD, blue = ipsilateral striatum, yellow = contralateral striatum. *, *P* < 0.05; **, *P* < 0.01; *post hoc* Tukey test (*n* = 5 per group). **G,** Graph showing volume of MPIO-induced hypointensities beyond the border of gadolinium enhancement for rats injected with either VCAM-MPIO or IgG-MPIO. *, *P* < 0.05; Student *t* test. **H,** Pseudocolored three-dimensional reconstruction of imaged animal brain, with coregistration of post-VCAM-MPIO *T*_2_*-weighted and contrast-enhanced *T*_1_-weighted images; blue = gadolinium enhanced region and yellow = *T*_2_* hypointensities. **I,** Representative *T*_2_*-weighted MR image slice of an U87MG tumor, with white box highlighting the invasive tumor margin confirmed histologically (**J**); scale bar = 500 μm. **K,** Corresponding high-power magnification of highlighted regions of interest (black and red boxes) from stained adjacent sections, demonstrating bound VCAM-MPIO beads (black arrowhead/brown spheres) in a VCAM-1–positive vessel lumen (brown); scale bar = 10 μm.

In contrast to the MDA231Br-GFP tumors, post-gadolinium contrast enhancement was present on *T*_1_-weighted images for all the U87MG intrastriatal tumors (Fig. 4A and B). As for the metastasis animals, U87MG mice showed significantly increased volumes of hypointensity in the left striatum following intravenous injection of VCAM-MPIO, with minimal hypointensities present in the contralateral striatum (Fig. 4C and E). No increase in hypointensities on *T*_2_*-weighted images was evident in tumor-bearing animals injected with IgG-MPIO (Fig. 4D and E). Compared with post-gadolinium *T*_1_-weighted images, VCAM-MPIO induced hypointensities were visible at sites beyond the edge of gadolinium-induced hyperintensity (Fig. 4F). Furthermore, quantitative assessment showed a significantly greater volume of hypointensities extending beyond the area of gadolinium enhancement (*P* < 0.05), compared to tumor-bearing rats injected with IgG-MPIO (Fig. 4G), which visually represented the infiltrative tumor margin (Fig. 4H).

Subsequent, histologic analysis of the U87MG tumors demonstrated specific binding of VCAM-MPIO on VCAM-1–expressing vessels in proximity to the edge of the tumor. The sites of invasive U87MG tumor with VCAM-MPIO binding correlated with the hypointense signal visualized on *T*_2_*-weighted MRI (Fig. 4I–K).

**Figure 5.**
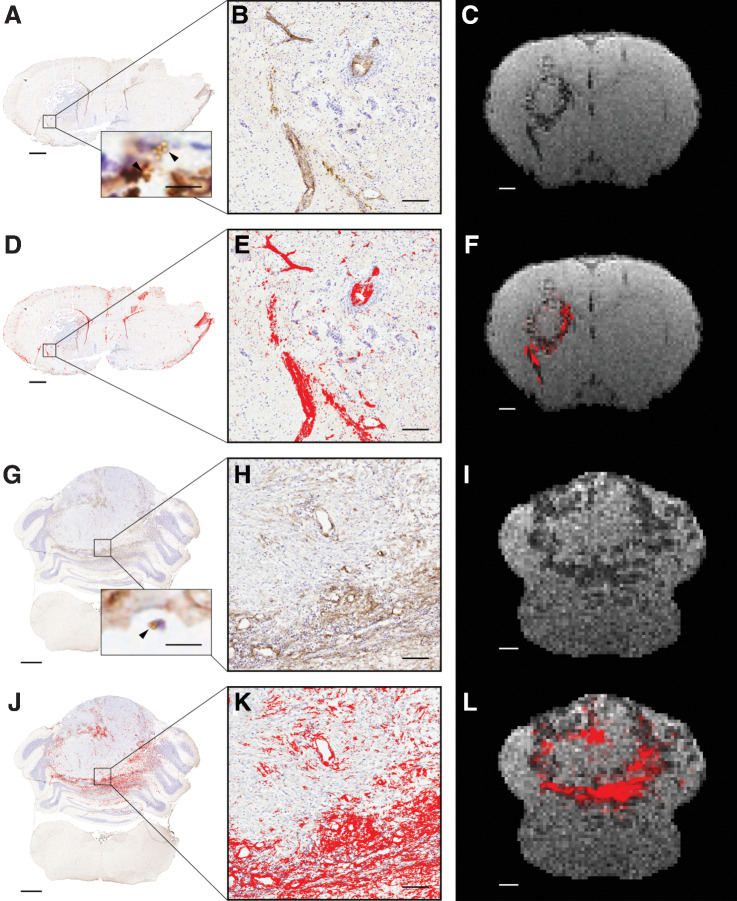
Spatial colocalization of VCAM-1 and VCAM-MPIO–induced hypointensities in metastasis and medulloblastoma models. Representative images from a MDA231Br-GFP tumor-bearing animal (**A–F**) and a DAOY medulloblastoma animal (**G–L**). Positive VCAM-1 expression (brown) is evident on the endothelium of vessels at the margins of both the MDA231Br-GFP tumor (**A** and **D**) and the DAOY tumor (**G** and **J**); cresyl violet counterstain (blue). Scale bar = 1 mm. Inset images in **A** and **G** illustrate presence of VCAM-MPIO beads (black arrowhead) bound to the luminal surface of VCAM-1–positive vessels (brown); scale bar = 10 μm. **B**, **E**, **H**, and **K**, Corresponding high-powered magnification images from boxes; scale bar = 100 µm. **D**, **E**, **J**, and **K**, Areas segmented as VCAM-1 positive are highlighted in red. Note, in **A** and **D** nonspecific staining of the ventricles and tissue edges is evident, which has not been removed from the analysis for transparency, but does not reflect true endothelial VCAM-1 staining. **C**, **F**, **I**, and **L**, Corresponding *T*_2_*-weighted MRI slices showing VCAM-MPIO–induced hypointensities either alone (**C** and **I**), or with an overlay (red) showing perspective transformed histology derived VCAM-1 staining (**F** and **L**); scale bar = 1 mm.

Spatial colocalization of the VCAM-MPIO–induced hypointensities and endothelial VCAM-1 staining was further assessed in a MDA231Br-GFP injected rat, by converting the VCAM-1 IHC staining map to MRI resolution and overlaying on the T_2_*-weighted image (Fig. 5A–F). In addition, further validation of the applicability of the VCAM-MPIO agent was demonstrated in an orthotopic DAOY medulloblastoma rat model (Fig. 5G–L). In both models, the overall histologic staining pattern of endothelial VCAM-1 was congruous with the distribution of VCAM-MPIO–induced MRI hypointensities (Fig. 5F and L). Moreover, for both metastasis and medulloblastoma models, significantly greater histologic VCAM-1 staining intensity was evident in regions of MRI-visible hypointensities than in regions without hypointensities (Kruskal–Wallis and *post hoc* Dunn test, *P* < 0.001 for both).

### The peritumoral region of human brain metastases is associated with upregulated endothelial VCAM-1 expression

**Figure 6.**
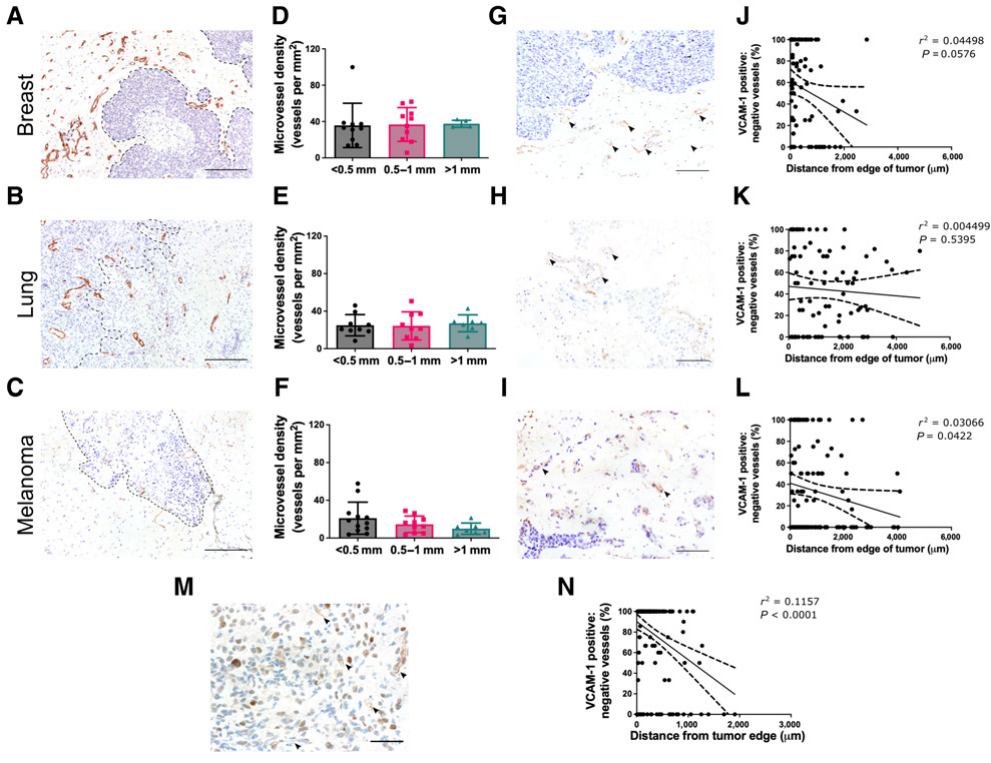
Peritumoral vasculature is characterized by upregulated endothelial VCAM-1 expression. Representative histologic sections of peritumoral brain tissue, from breast cancer (**A**), lung adenocarcinoma (**B**), and melanoma brain metastases (**C**) immunohistochemically stained for the endothelial marker, CD34 (brown); scale bar = 200 μm, black dashed line outlines tumor border. **D–F,** Corresponding graphs showing mean microvessel density (±SD) in the peritumoral brain parenchyma in the following regions: < 0.5 mm, 0.5–1 mm, and > 1 mm from the metastasis border; *n* = 10 (breast), *n* = 9 (lung), and *n* = 12 (melanoma). **G–I,** Images showing endothelial VCAM-1 expression (arrowheads) associated with the metastasis from the same patients as above; scale bar = 100 μm. **J–L,** Corresponding graphs showing ratio of VCAM-1–positive/negative vessels in randomly distributed square ROIs across the peritumoral brain parenchyma. **M,** Representative image of endothelial VCAM-1 expression at the border of a glioblastoma, with corresponding graph of VCAM-1–positive/negative vessel distribution (**N**); scale bar = 100 μm.

Microvessel density in the peritumoral brain in association with the brain metastasis was heterogeneous across the different primary tumor types; the mean number of peritumoral vessels per mm^2^ (±SD) was 33.2 ± 15.1 for breast cancer, 24.3 ± 10.5 for lung adenocarcinoma, and 16.8 ± 9.5 for melanoma. In the peritumoral brain parenchyma, no statistical differences in microvessel density, according to distance from the tumor border, were found for any of the tumor types (Fig. 6A–F).

Analysis of endothelial VCAM-1 distribution indicated a greater concentration of VCAM-1–positive vessels in proximity to the tumor edge (Fig. 6G–L). Where VCAM-1 expression was visible further from the tumor border, adjacent sections stained for tumor-specific markers often revealed isolated tumor cells or tumor clusters nearby (Supplementary Fig. S6). In the melanoma brain metastasis samples, a statistically significant inverse relationship was evident between the concentration of VCAM-1–positive vessels and distance from the tumor border (*P* = 0.0422; Fig. 6L). While the breast cancer and lung adenocarcinoma brain metastasis samples showed similar trends, these did not reach statistical significance, albeit only just for the breast cancer cohort (*P* = 0.0576; Fig. 6J). In the glioblastoma samples, the number of VCAM-1–positive vessels significantly increased with closer proximity to the tumor border (Fig. 6M and N); although the area of adjacent “uninvolved” brain parenchyma was more limited in the samples, in comparison with the brain metastasis samples.

Analysis of the corresponding human MRI data revealed notable limitations. For post-gadolinium *T*_1_-weighted imaging, no significant differences were found between signal intensities in the tumor border (i.e., brain tissue immediately adjacent to the area of contrast enhancement or tumor core) and peritumoral brain tissue, for any of the tumor types (Supplementary Fig. S7). Thus, these conventional MRI modalities did not confer a consistent or definitive signature for either the tumor border or adjacent “uninvolved” brain tissue despite the presence of vascular inflammation and small tumor clusters in these regions, as indicated above. These data indicate that the endothelial VCAM-1 upregulation observed histologically at the invasive tumor margin extends beyond the apparent tumor border, which was not realized radiologically on either gadolinium contrast-enhanced or diffusion-weighted MRI (Supplementary Fig. S7).

## Discussion

Local therapies, such as surgical resection and focal radiotherapy, are a key part of the treatment strategy for primary and metastatic brain tumors, but effective application relies on accurate delineation of the tumor margins. At the same time, owing to the eloquent nature of certain regions in the brain, sparing of uninvolved brain tissue is vital to minimize neurologic deficits. Therefore, accurate knowledge of the extent of the tumor margin is required for optimal treatment planning (18). Here, we demonstrate that a VCAM-1–targeted imaging approach has the potential to better detect brain tumor margins, using preclinical models of breast cancer brain metastasis and glioblastoma, than currently used clinical MRI methods. Furthermore, we extend these findings to an orthotopic model of medulloblastoma, illustrating the broader potential of this approach for other types of primary brain tumors. Finally, we show that VCAM-1 is upregulated at the invasive margins of human brain metastasis and glioblastoma samples and extends beyond the tumor margin defined by conventional radiological methods, supporting the potential clinical utility of this approach.

Advanced noninvasive imaging has become a necessary tool in both surgical and radiotherapy planning. MRI is the preferred imaging modality for detailed anatomic characterization in the brain, and in brain cancer the current gold standard relies on extravasation of gadolinium-based paramagnetic contrast agents across a disrupted blood–brain barrier (BBB) to delineate tumor extent on *T*_1_-weighted images (19). The tumor edge, however, is an ill-defined region, which may include isolated tumor cells or tumor clusters that are discontinuous from the main tumor bulk. Conventionally, metastatic brain tumors have been considered to show limited invasion into the adjacent brain parenchyma. However, a number of studies have now shown that human metastatic brain tumors can display a range of invasive phenotypes microscopically, with variation in the depth of infiltration depending on tumor type (2, 3, 20). Moreover, frequent local tumor recurrence, within the surgical or radiotherapy treatment field, points to the presence of residual microscopic disease despite radical macroscopic treatment. For primary brain tumors, infiltration of tumor cells into surrounding white matter is a hallmark of the disease (21). Importantly, however, in glioblastoma, it is believed that, whilst the BBB is permeable within the main tumor mass, it remains intact at the infiltrative edge. The biology of the invasive margin for brain metastases has been less well studied. However, it has been reported that the BBB in brain metastases is selectively disrupted, resulting in heterogeneous permeability that may impair the penetration of chemotherapeutic agents (22, 23) or imaging contrast agent extravasation. Thus, incomplete BBB permeability throughout the tumor, and particularly at the tumor-brain interface will compromise accurate tumor delineation by conventional contrast-enhanced MRI.

We have previously shown that VCAM-1–targeted MRI enables early detection of micrometastases in the brain (11, 12), and that preliminary data in human samples of brain metastases indicated VCAM-1 upregulation at the invasive margin. From these observations, we proposed that this technique might also enhance detection of the tumor-brain interface of more advanced brain tumors. To test this hypothesis, we have used xenograft models, induced through direct intracerebral injection of human-derived MDA231Br-GFP or U87MG tumor cells. The U87MG cell line has been well characterized as a model for human glioblastoma and has been shown to display a distinct invasive pattern in *in vitro* systems, with prominent cellular protrusions (24). However, the limitations of the U87MG model *in vivo* have also been widely reported, as it lacks a diffusely infiltrative cellular pattern at the tumor margin and, therefore, does not fully replicate the tumor-brain interface typically seen in human glioblastoma (25). In the current study, however, we observed that the U87MG intracranial model produced highly proliferative tumors that had a disrupted tumor border, with finger-like projections and isolated tumor clusters separated from the tumor bulk. Importantly, even with a less infiltrative pattern of invasion, we demonstrated that the VCAM-MPIO imaging was able to detect the tumor-brain interface more sensitively than gadolinium contrast-enhanced MRI. Moreover, the presence of upregulated endothelial VCAM-1 in close association with the tumor front in human glioblastoma tissue provides confidence that VCAM-MPIO MRI remains relevant in more infiltrative disease. In contrast, the MDA231Br-GFP tumor model was characterized by vascular cooption of tumor cells at the tumor-brain interface, which is consistent with clinical observations of heterogeneous invasion patterns seen in brain metastatic disease (2). Thus, we have shown that VCAM-MPIO MRI can improve the detection of the tumor-brain interface across a broad range of invasive patterns, which is necessary for more intelligent delineation of treatment planning.

In keeping with previously published work, microvessel density was found to be significantly greater at the tumor periphery than in the tumor core, or in the normal brain tissue (26). Moreover, endothelial VCAM-1 was markedly upregulated at the tumor margins in both models, with negligible expression evident in normal brain tissue, as previously reported (11, 12). Upregulation of both Ki67 and SOX2 within the same marginal areas of the tumors is indicative of a highly proliferative and invasive tumor cell phenotype and suggests that VCAM-1 upregulation is closely associated with the invasive front of the tumor. These findings support the hypothesis that VCAM-1–targeted imaging may provide a sensitive biomarker for brain tumor margins that is not currently provided by clinical imaging methods.

Subsequent *in vivo* VCAM-1–targeted imaging studies demonstrated that, despite no evident gadolinium contrast enhancement, VCAM-MPIO induced hypointensities were clearly visible in the brain metastasis model and revealed areas of tumor that were also not detectable by the alternative conventional clinical method of *T*_2_-weighted MRI. Lack of gadolinium contrast enhancement suggests an intact BBB likely due to the vascular co-optive growth pattern of MDA231Br-GFP tumors despite their relatively advanced stage, as demonstrated histologically. For the glioblastoma model, the region of VCAM-MPIO–induced hypointensities extended beyond the boundaries of the tumor indicated through gadolinium enhanced *T*_1_-weighted MRI. In both models, histologic examination confirmed that VCAM-MPIO were localized to VCAM-1–positive vessels at the tumor margins. The spatial correspondence of VCAM-1 upregulation and the VCAM-MPIO–induced hypointensities was further confirmed in both the brain metastasis model and an orthotopic model of medulloblastoma, supporting the broader applicability of this concept across multiple brain tumor types.

Using spatially coregistered brain tissue samples from the tumor-brain interface, we interrogated the microstructural properties of the brain parenchyma directly adjacent to human brain metastases and glioblastoma. The availability of linked human imaging data has allowed us to correlate the MRI findings with the “gold standard” technique for detecting the tumor-brain interface, that is, histology. Here, we have shown that endothelial expression of VCAM-1 is upregulated in close proximity to the tumor and, particularly, at the tumor-brain interface. Moreover, where endothelial VCAM-1 expression was more distant from the tumor mass, these vessels were associated with microscopic disease that would have been undetectable with standard gadolinium contrast-enhanced MRI. The significance of endothelial VCAM-1 upregulation at the invasive margin is further emphasized by the lack of objective demarcation from normal brain tissue on conventional contrast-enhanced *T*_1_-weighted and diffusion-weighted MRI.

In accord with our findings, previous studies investigating the correlation between image-guided stereotactic biopsies and abnormalities defined on conventional MRI found that isolated infiltrating glioma cells were present even at sites without *T*_1_- and *T*_2_-weighted prolongation. Conversely, where there was *T*_1_- and *T*_2_-weighted prolongation, negative biopsies corresponding to edematous tissue without tumor infiltration were also obtained (6, 7, 27). Our findings are also consistent with a previous study showing that ADC values do not significantly vary according to distance from the peritumoral region of brain metastases (4). Similarly, a considerable overlap in ADC values between glioma and peritumoral brain tissues has been shown (28), suggesting that ADC values may not be a reliable marker of the invasive tumor edge. It is apparent, therefore, that standard MRI techniques have inadequate sensitivity and specificity to accurately differentiate the invasive margin from surrounding brain parenchyma.

Together, these preclinical and clinical data suggest that VCAM-1–targeted MRI has the potential to augment existing MRI techniques, by exploiting microenvironmental changes at the periphery of brain tumors for greater accuracy of margin delineation, despite an undisrupted BBB. Tumor infiltration into the brain parenchyma is already well recognized in primary brain malignancies and we present here evidence that this clinical problem is also relevant in brain metastases. This phenomenon currently poses the greatest diagnostic challenge to conventional gadolinium-enhanced MRI and in treatment planning/delivery. However, MRI is not limited to a single modality and, in fact, allows a multitude of sequences to be performed within a single-imaging session. We have previously demonstrated that combining multiple MRI modalities can improve delineation of true tumor extent in the brain (29). The results of the current study lend further support to the clinical development of VCAM-targeted MRI for inclusion in such multiparametric imaging paradigms, potentially leading to significant improvements in the management of both primary and secondary brain tumors.

## Supplementary Material

Supplementary Figure

Supplementary Figure

Supplementary Figure

Supplementary Figure

Supplementary Figure

Supplementary Figure

Supplementary Figure

Supplementary Data

Supplementary Table

Supplementary Table
