# Supplementary material for: VCAM-1–targeted MRI Improves Detection of the Tumor-brain Interface
Source: Clin Cancer Res. 2022 Mar 1;28(11):2385–96. doi: 10.1158/1078-0432.CCR-21-4011 (PMC9662863; doi:10.1158/1078-0432.CCR-21-4011)
Supplement: Supplementary Figure [file ccr-21-4011_figure_s1_supps1.pdf]

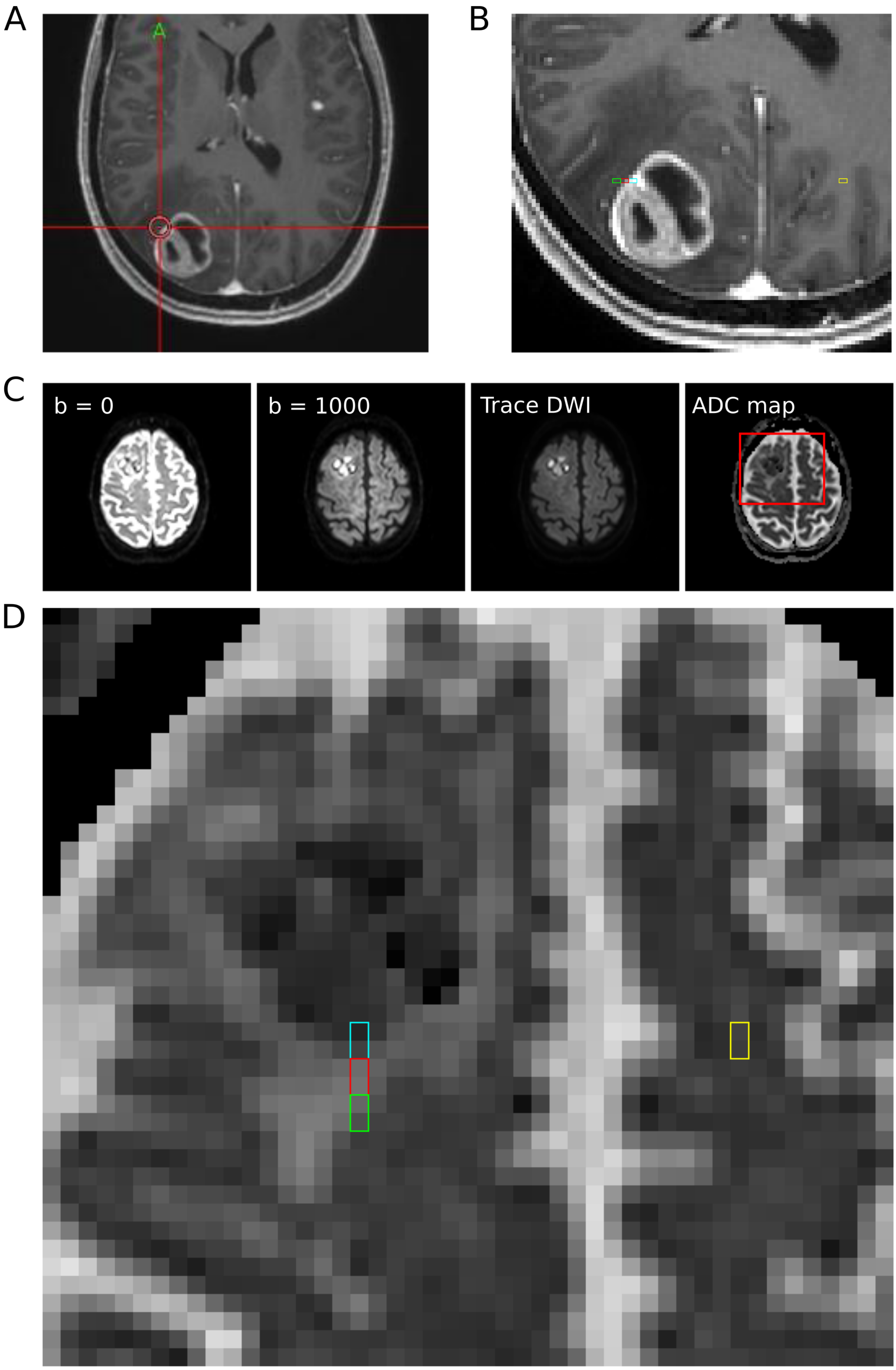
A
B
C
b = 0
b = 1000
Trace DWI
ADC map
D

Fig S1. Analysis of human MRI at site of stereotactic biopsy. (**A**) An example post-gadolinium T1-weighted axial MR image of a human subject demonstrating a right occipital enhancing lesion, in keeping with a melanoma brain metastasis. Red marker indicates the site of the stereotactic biopsy, targeting the brain metastasis-brain parenchyma interface, collected under image guidance, as described by Zakaria *et al*[1]. (**B**) Specific regions of interest, marking the tumor core (cyan rectangle), tumor rim (red rectangle), adjacent parenchyma (green rectangle) and contralateral brain (yellow rectangle), were used to measure the mean grey pixel intensity corresponding to the biopsy location. (**C**) Example diffusion-weighted axial MR images of a human subject demonstrating a right frontal enhancing lesion, in keeping with a lung adenocarcinoma brain metastasis. Images were taken at b = 0 and 1000 s/mm^2. A trace diffusion-weighted image (DWI) was calculated as the mean of the various gradients, from which an apparent diffusion coefficient (ADC) map was derived. (**D**) At the site of the tumor (red box), specific regions of interest, marking the tumor core, tumor rim, adjacent parenchyma and contralateral brain, were used to measure the ADC values corresponding to the biopsy location.
