# Supplementary material for: VCAM-1–targeted MRI Improves Detection of the Tumor-brain Interface
Source: Clin Cancer Res. 2022 Mar 1;28(11):2385–96. doi: 10.1158/1078-0432.CCR-21-4011 (PMC9662863; doi:10.1158/1078-0432.CCR-21-4011)
Supplement: Supplementary Figure [file ccr-21-4011_figure_s2_supps2.pdf]

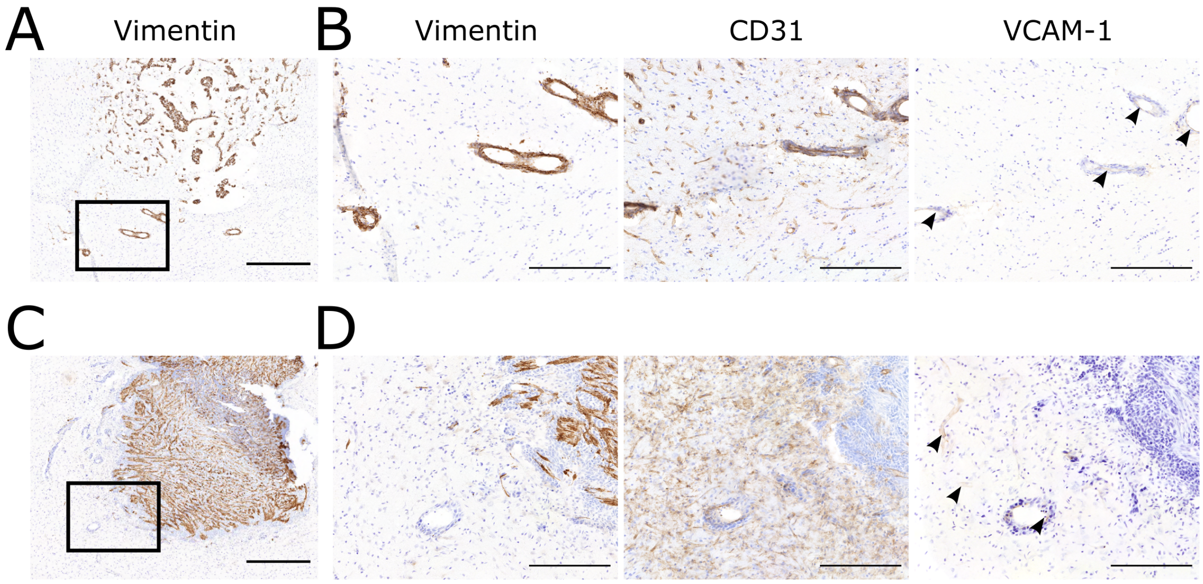

Fig S2. Both MDA231Br-GFP and U87 intracranial tumors demonstrate regions with a poorly demarcated border. **(A)** Example digital micrograph of a MDA231Br-GFP intracranial tumor, stained for vimentin (brown color), with an infiltrative border at the brain tumor-brain parenchyma interface. Scale bar = 500 µm; Black window highlights clusters of tumor cells that have escaped from the main tumor mass. **(B)** Higher powered magnification digital micrographs of the irregular border in serial tissue sections illustrate the specific staining (brown color) for vimentin (tumor cells), CD31 (blood vessels) and endothelial VCAM-1 (arrowheads). Scale bar = 200 µm. **(C-D)** A similar observation was seen with the U87MG model.
