# Supplementary material for: VCAM-1–targeted MRI Improves Detection of the Tumor-brain Interface
Source: Clin Cancer Res. 2022 Mar 1;28(11):2385–96. doi: 10.1158/1078-0432.CCR-21-4011 (PMC9662863; doi:10.1158/1078-0432.CCR-21-4011)
Supplement: Supplementary Figure [file ccr-21-4011_figure_s3_supps3.pdf]

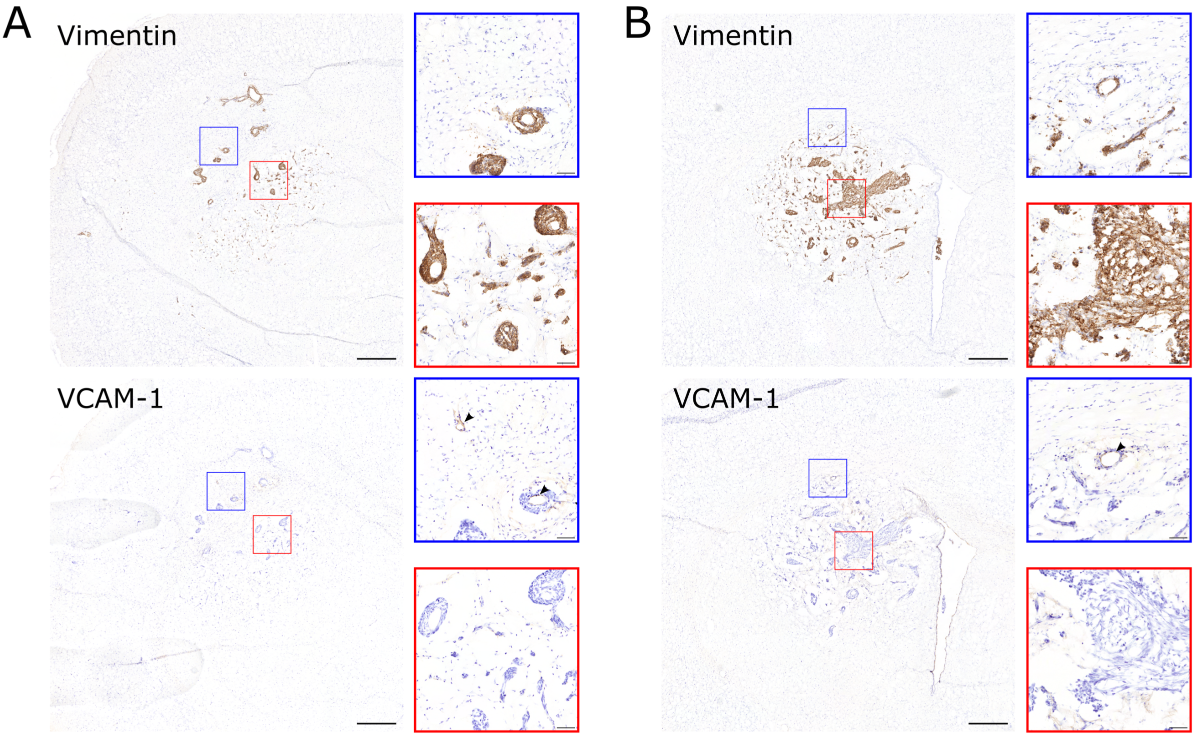


**Fig S3. Endothelial VCAM-1 is associated with the tumor invasive rim in MDA231Br-GFP models.** Brightfield digital micrographs of MDA231Br-GFP tumor sections in mouse brain immunohistochemically stained (brown) for either Vimentin (tumor cells) or VCAM-1 (endothelial cells; black arrowheads), corresponding to animals from Fig 3A (**A**) and Fig 3B (**B**). Scale bar = 500 µm. Blue and red boxes correspond to high-power magnification images of the tumor rim and tumor core, respectively. Scale bar = 50 µm.
