# Supplementary material for: VCAM-1–targeted MRI Improves Detection of the Tumor-brain Interface
Source: Clin Cancer Res. 2022 Mar 1;28(11):2385–96. doi: 10.1158/1078-0432.CCR-21-4011 (PMC9662863; doi:10.1158/1078-0432.CCR-21-4011)
Supplement: Supplementary Figure [file ccr-21-4011_figure_s4_supps4.pdf]

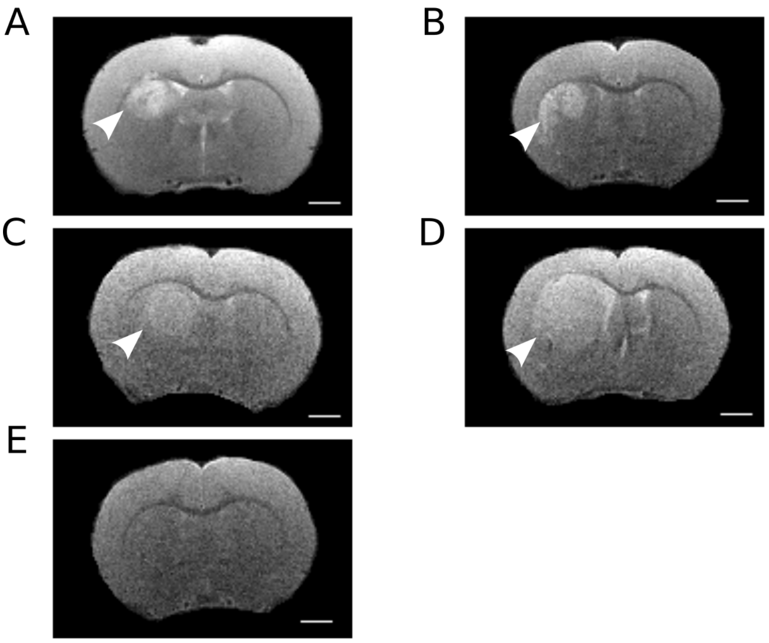

Fig S4. T_2-weighted MRI permits tumor localization. Corresponding T_2-weighted images for MDA231Br-GFP tumor bearing rat brains from Fig 3, prior to (**A**) VCAM-MPIO and (**B**) IgG-MPIO administration. Corresponding T_2-weighted images for U87MG tumor bearing rat brains from Fig 4, prior to (**A**) VCAM-MPIO and (**B**) IgG-MPIO administration. (**E**) Corresponding T_2-weighted image for PBS injected rat brain from Fig 3C. White arrowhead denotes location of tumor; scale bar = 2 mm.
