# Supplementary material for: VCAM-1–targeted MRI Improves Detection of the Tumor-brain Interface
Source: Clin Cancer Res. 2022 Mar 1;28(11):2385–96. doi: 10.1158/1078-0432.CCR-21-4011 (PMC9662863; doi:10.1158/1078-0432.CCR-21-4011)
Supplement: Supplementary Figure [file ccr-21-4011_figure_s5_supps5.pdf]

A

B

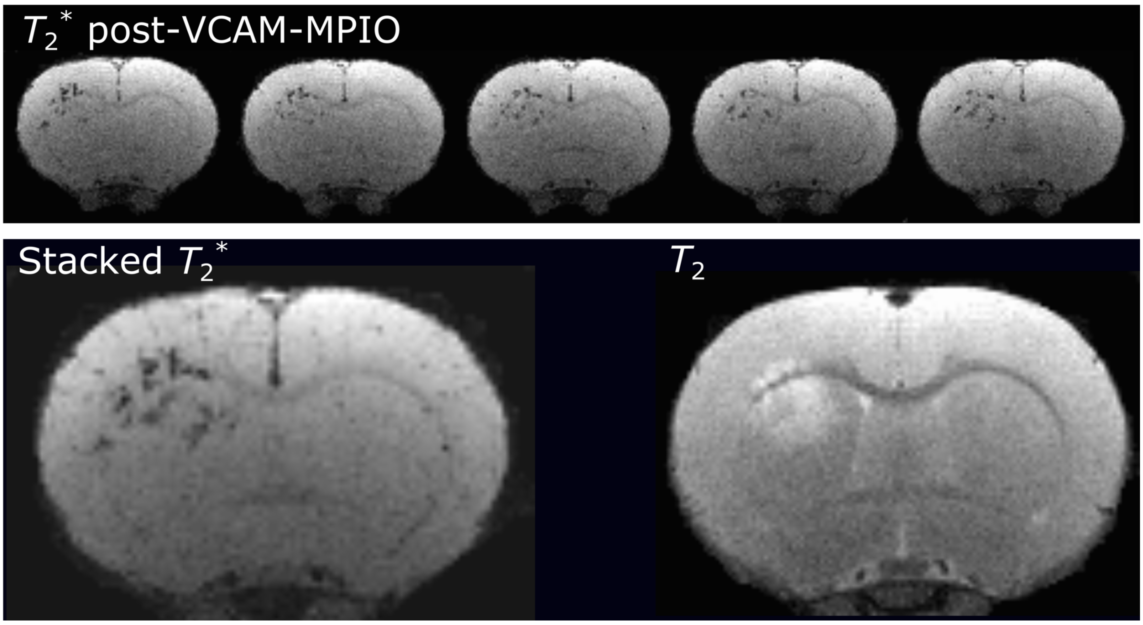

Fig S5. VCAM-MPIO signal encompasses the tumor perimeter on *T_2^**-weighted MRI. (**A**) T_2^*-weighted MR images (slice thickness 120 μm each) following VCAM-MPIO administration of a mouse brain containing an MDA231Br-GFP tumor, which have been (**B**) stacked to form a composite image and compared side-to-side with the corresponding T_2-weighted MR image from Fig 3G (slice thickness 500 μm).
