# Supplementary material for: VCAM-1–targeted MRI Improves Detection of the Tumor-brain Interface
Source: Clin Cancer Res. 2022 Mar 1;28(11):2385–96. doi: 10.1158/1078-0432.CCR-21-4011 (PMC9662863; doi:10.1158/1078-0432.CCR-21-4011)
Supplement: Supplementary Figure [file ccr-21-4011_figure_s6_supps6.pdf]

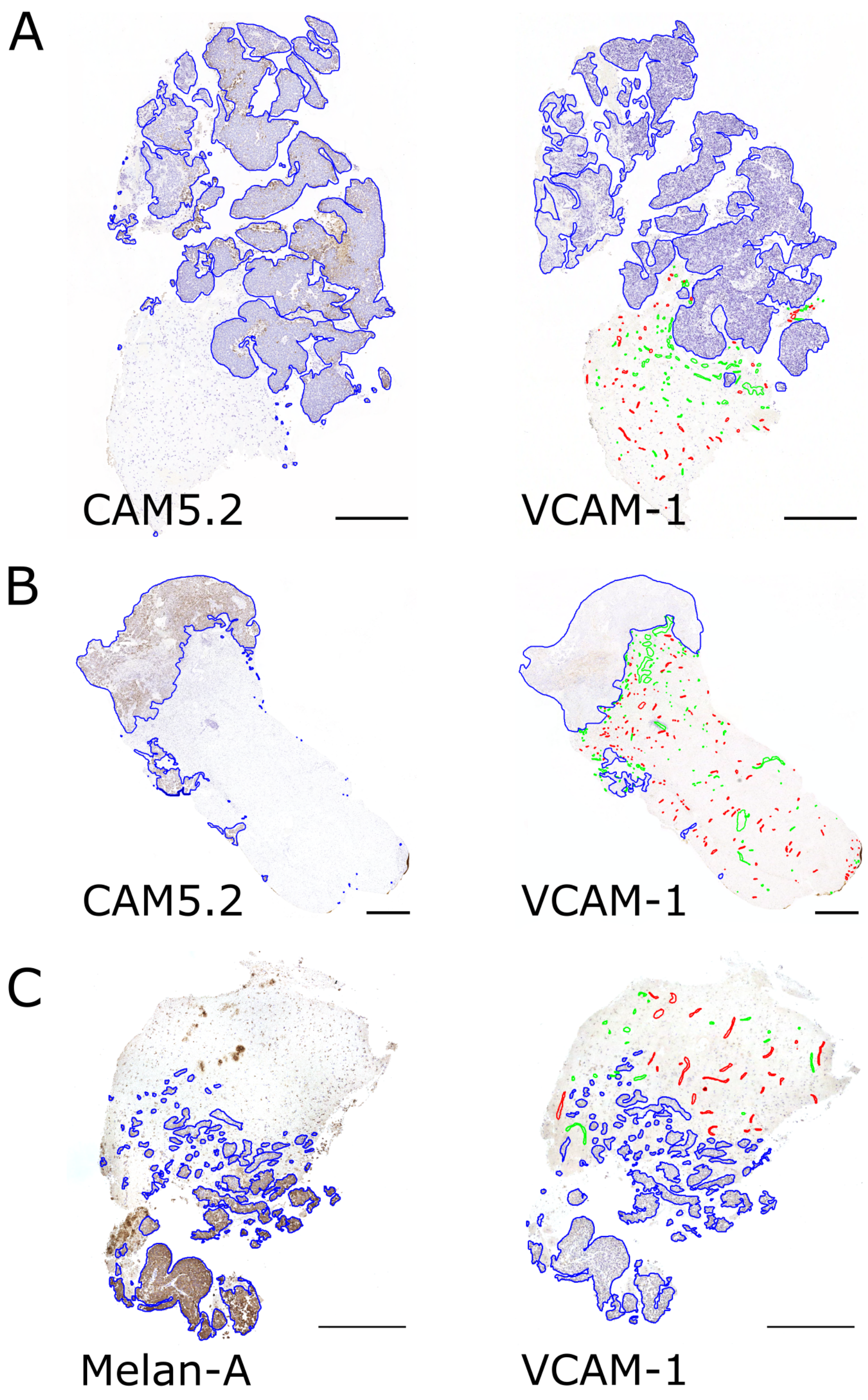


**Fig S6. Distribution of endothelial VCAM-1 staining in relation to brain metastasis margin.** Representative histological sections of human brain tissue containing metastases from (**A**) breast cancer, (**B**) lung adenocarcinoma and (**C**) melanoma primaries comparing the distribution of endothelial VCAM-1 in relation to the location of tumor cells; scale bar = 500

μm for breast and melanoma, and 1 mm for lung adenocarcinoma. Sections have been immunohistochemically stained for a tumor-specific marker – either CAM5.2 or melan-A (tumor area outlined in blue). Adjacent sections were immunohistochemically stained for VCAM-1; corresponding tumor area has been outlined in blue, VCAM-1 positive vessels outlined in green and VCAM-1 negative vessels outlined in red.
