# Supplementary material for: VCAM-1–targeted MRI Improves Detection of the Tumor-brain Interface
Source: Clin Cancer Res. 2022 Mar 1;28(11):2385–96. doi: 10.1158/1078-0432.CCR-21-4011 (PMC9662863; doi:10.1158/1078-0432.CCR-21-4011)
Supplement: Supplementary Figure [file ccr-21-4011_figure_s7_supps7.pdf]

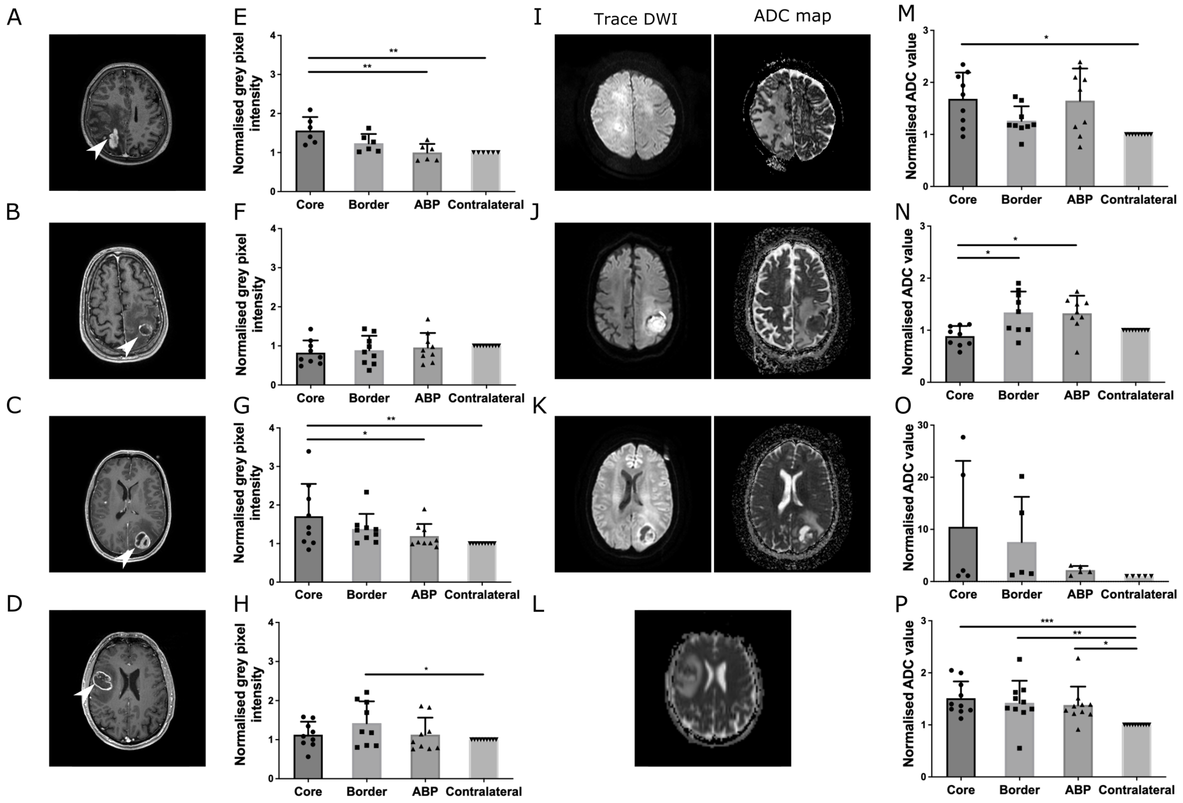

Fig S7. Gadolinium contrast enhanced T_1-weighted and diffusion-weighted MRI measurements at the brain metastasis and glioblastoma border. (**A-D**) T_1-weighted MR images, following i.v. gadolinium administration, of human patients with brain metastasis, from (**A**) breast cancer, (**B**) lung adenocarcinoma and (**C**) melanoma, and (**D**) glioblastoma. (**E-H**) Corresponding graphs showing mean pixel signal intensity (± standard deviation; SD) at the tumor core, tumor border and adjacent brain parenchyma (ABP) normalized to the contralateral brain; n = 6 (breast), n = 8 (lung), n = 9 (melanoma) and n = 9 (glioblastoma). (**I-L**) Diffusion-weighted images (DWI) from the same patients as above, and the corresponding apparent diffusion coefficient (ADC) maps generated from the diffusion-weighted images. ADC maps alone was generated for the glioblastoma cohort. (**M-P**) Corresponding graphs showing mean ADC values (± SD) at the tumor core, tumor border and ABP. *$p<0.05$, **$p<0.01$; post-hoc Tukey multiple comparisons test. White arrowhead denotes location of tumor.
