# Supplementary material for: VCAM-1–targeted MRI Improves Detection of the Tumor-brain Interface
Source: Clin Cancer Res. 2022 Mar 1;28(11):2385–96. doi: 10.1158/1078-0432.CCR-21-4011 (PMC9662863; doi:10.1158/1078-0432.CCR-21-4011)
Supplement: Supplementary Data [file ccr-21-4011_supplementary_methods_suppsm.pdf]

Supplementary methods

Rat histology analysis

Rat brain sections (*ca*. 20 per animal) were immunohistochemically stained for markers to define the tumor cells (vimentin), endothelium (CD31), endothelial VCAM-1 expression, tumor cell stemness (nestin, SOX2) and cellular proliferation (Ki67). Stained tissue sections were digitally scanned on the Aperio CS2 machine, at x20 magnification, with the images stored offline for analysis.

The digital images were visualized using ImageScope software and manually annotated to highlight the following regions of interest (ROIs): tumor core, defined as 90% of the tumor bulk, excluding any brain parenchyma; tumor rim, defined as the remaining tumor periphery; and the contralateral striatum. Using the in-built Positive Pixel Count v.9 algorithm, all medium and strong intensity brown pixels (Intensity threshold between 0 to 174, intensity threshold of negative pixels = -1, Color saturation threshold = 0.04, Hue width = 0.5) were summed for each section and divided by the total area of the corresponding ROI to determine the relative staining of each marker, reported as area of positive pixels per mm^2.

Antibody-MPIO synthesis

Conjugation of antibodies to the outer coating of MPIOs was performed as previously described[2]. Briefly, 5 mg of 1 μm diameter MPIO (Dynabeads MyOne Tosylactivated) were suspended in equal measures of 0.1M sodium borate buffer, 3M ammonium sulphate buffer and 0.5 mg/mL antibody, either VCAM-1 (cat. 14-1060-85; eBioscience) or mouse IgG isotype control (cat. E06660-1641; eBioscience). The MPIO suspension was incubated for 24 h at 37°C under continuous agitation at 1000rpm. The following day, the beads were pelleted in a magnet, the supernatant removed and replaced with 0.5% w/v bovine serum albumin (BSA) in PBS-T,

before another incubation period as per previous step. On the final day, the MPIO beads were pelleted and sequentially washed in 0.1% w/v BSA in PBS-T three times, before storage in 200 μL of 0.1% w/v BSA in PBS. Successful antibody loading (~20,000 antibodies per MPIO) was confirmed via fluorescence-activated cell sorting, compared against standardized calibration beads.

Animal MRI sequences

Prior to image acquisition for each animal, inhomogeneities in the main magnetic field (B0) were corrected through active shimming of the coils. Subsequently, a set of 24 axial T_2-weighted images (slice thickness = 0.5 mm) was acquired using a 2D fast spin-echo sequence (TR = 3000 ms, TE = 35.65 ms, matrix size = 256 × 256, number of slices (NS) = 24, NT = 2, FOV = 32 mm × 32mm). For VCAM-MPIO detection, a T_2^*-weighted 3D gradient echo dataset (GE3D) was acquired pre- and post-MPIO administration with the following parameters: flip angle 27°, TR = 65 ms, TE = 7.5 ms, FOV = 32 mm x 32 mm x 16 mm, matrix size = 256 x 192 x 96, NT = 2 and total acquisition time ~40 mins. The mid-point of acquisition was 1 ± 0.2 h after MPIO injection. Finally, the animal underwent T_1-weighted imaging using a GE3D sequence, pre- and 5 mins post-intravenous gadolinium-DTPA injection (30 μL), to identify BBB permeability with the following parameters: flip angle 10°, TR = 5.38 ms, TE = 2.71 ms, FOV = 32 mm x 32 mm x 16 mm, matrix size = 256 x 192 x 96, NT = 8 and total acquisition time ~13 mins. Data were zero-filled to 256 x 256 x 256, to a final isotropic resolution of 120 μm.

Animal MR image processing

Each dataset of T_2^*-weighted images was manually masked and segmented to exclude extracerebral structures using ITK-SNAP[3]. Automated image processing of segmented images

was performed using a custom designed MATLAB code[4]. Briefly, hypointense signals were defined as a voxel value 0.65 times less than the mean value. Signals arising from ventricles or sinuses, which appear hypointense naturally, were excluded by imposing an upper threshold size limit of 20 voxels. A lower threshold filter of 1 voxel size was used to exclude noise. The threshold cut-offs and automated analysis were optimized in prior work to enable a detection rate of 98.3 ± 0.49% of total brain hypointensities[4]. An additional manual clean was performed to exclude extraneous hypointensities arising from normal vascular territories and the cerebellum. Segmented images were reconstructed to visualize the spatial distribution of MPIO binding. Voxel volumes were summed and expressed as raw volumes in microliters.

T_1-weighted post-gadolinium (Gd) image sets were co-registered with T_2^*-weighted post-MPIO image sets. Masks delineating the extent of post-Gd T_1 hyperintensity were prepared by visual inspection and masks of left and right hemispheres were prepared by bisecting the whole-brain mask along the midline. For all animals, the volume of hypointensities was summed for both the ipsi- and contralateral hemispheres. The MDA231Br and U87MG tumor-bearing animals, hypointensity masks were overlaid with post-Gd T_1-weighted masks to determine volumes of hypointensities both within and without the Gd-enhancing area. All analyses were performed using MATLAB R2019a (MathWorks, Natick MA, USA).

Co-registration analysis of MRI hypointensities and tissue VCAM-1 expression

For co-registration of histology and MRI findings, scanned images of histological sections were imported into ImageJ as 8-bit RGB images. Areas positive for VCAM-1 staining were selected by thresholding with hue either <114 or >216 and saturation > 25. Histology sections were matched to MRI slices, first by reference to anatomical features, shape, and a brain atlas

before being aligned accurately using a manual perspective transform, by applying stretch and/or image distortion, to account for tissue warping and/or shrinkage.

Human histology analysis

The digital images were visualized using ImageScope (v12.3.3, Leica) and manually annotated to highlight the following ROIs: tumor and VCAM-1 positive vessels. To determine the relationship between endothelial VCAM-1 activation and proximity to tumor, standard 100 μm x 100 μm square ROIs were randomly dispersed across the expanse of the brain parenchyma and the number of VCAM-1 positive vessels within each ROI was quantified, expressed as positive vessels per mm^2. Microvessel density was quantified based on number of CD31 stained vessels within three specified ROI of peri-tumoral brain: within 500 μm, between 500 μm to 1 mm, or more than 1 mm distance from the tumor border.

Human MRI sequences

For the brain metastasis patients, images were acquired on a Philips Achieva 3T MRI scanner with standard head coils. T_1-weighted imaging was acquired using a fast spoiled gradient echo sequence, pre- and post-i.v. Gd administration with the following parameters: TR = 9 ms, TE = 1.4 ms, flip angle = 15°, matrix size = 256 x 256, number of slice = 180, slice thickness = 1 mm. Diffusion weighted imaging was acquired using a single-shot echo planar imaging with two b values of 0 and 1000 s/mm^2 with the following parameters: TR 2828 ms, TE 73 ms, matrix size = 128 x 128, slice thickness = 5 mm.

For glioblastoma imaging, a Siemens MAGNETOM Verio 3T MRI scanner was used with T_1-weighted imaging acquired using a magnetization-prepared rapid acquisition with gradient echo (MPRAGE) sequence pre- and post-i.v. Gd contrast administraton. The following

parameters were employed on the MPRAGE sequence: TR = 1900 ms, TE = 3.17 ms, flip angle = 9°, matrix size = 357 x 268, number of slice = 160, slice thickness = 1 mm. Diffusion weighted imaging was acquired using a single-shot echo planar imaging with two b values of 0 and 1500 s/mm^2 with the following parameters: TR 5900 ms, TE 84.8 ms, matrix size = 128 x 104, slice thickness = 2.5 mm.

Human MRI analysis

Where available, Digital Imaging and Communications in Medicine (DICOM) images relating to post-gadolinium T_1-weighted and diffusion-weighted sequences were selected for analysis. Post-gadolinium T_1-weighted DICOM images were analyzed using ImageJ (v1.51, NIH) and four specific regions of interest (ROIs), equally sized at 1 mm x 2 mm, were marked at the location of the tumor biopsy based on the tumor core, tumor rim, adjacent brain parenchyma and contralateral cortex (Fig S1A-B). The mean grey pixel intensity was measured. Diffusion-weighted DICOM images were accessed using DTIstudio (v2.0, John Hopkins University). As illustrated in Fig S1C, the DWI data were converted into an apparent diffusion coefficient (ADC) map using a pre-defined low angular gradient table. Based on the map, ADC values were measured using the same four specific ROIs, as previously mentioned.

Additional references

1. Zakaria R, Jenkinson MD. Using ADC Maps with Structural Scans to Improve Intraoperative Biopsy Specimens in Brain Metastases. *Neuroradiol J.* 2014; 27(4):422-424.

2. Serres S, Soto MS, Hamilton A, et al. Molecular MRI enables early and sensitive detection of brain metastases. *Proc Natl Acad Sci U S A.* 2012; 109(17):6674-6679.

3. Yushkevich PA, Piven J, Hazlett HC, et al. User-guided 3D active contour segmentation of anatomical structures: Significantly improved efficiency and reliability. *NeuroImage.* 2006; 31(3):1116-1128.

4. Hamilton A. *The effect of the systemic inflammatory response on the development of brain metastasis*. University of Oxford: Oncology, University of Oxford; 2013.
