# Supplementary material for: VCAM-1–targeted MRI Improves Detection of the Tumor-brain Interface
Source: Clin Cancer Res. 2022 Mar 1;28(11):2385–96. doi: 10.1158/1078-0432.CCR-21-4011 (PMC9662863; doi:10.1158/1078-0432.CCR-21-4011)
Supplement: Supplementary Table [file ccr-21-4011_table_s1_suppts1.docx]

| **Tumor histology** | ***n*** | **Tumor MRI volume (mean ± SD)** |
| --- | --- | --- |
| Breast cancer | 3 | 12.8 ± 9.5 mL |
| Lung adenocarcinoma | 3 | 4.7 ± 4.3 mL |
| Melanoma | 3 | 14.3 ± 3.8 mL |
| Glioblastoma | 7 | 19.9 ± 18.0 mL |

**Table S1. Radiological volume of human brain tumors based on gadolinium enhanced MRI.** Tumor volume measured on gadolinium contrast enhanced *T*_1_-weighted MRI for patients with a solitary brain tumor from either a breast cancer, lung adenocarcinoma or melanoma metastasis, or a glioblastoma. The low tumor volume of the lung adenocarcinoma brain metastases is accounted for by the significant cystic component of each lesion.
