# Supplementary material for: VCAM-1–targeted MRI Improves Detection of the Tumor-brain Interface
Source: Clin Cancer Res. 2022 Mar 1;28(11):2385–96. doi: 10.1158/1078-0432.CCR-21-4011 (PMC9662863; doi:10.1158/1078-0432.CCR-21-4011)
Supplement: Supplementary Table [file ccr-21-4011_table_s2_suppts2.docx]

| **Tumor histology** | ***n*** | **Mean biopsy length (± SD)** | **Max. biopsy length** | **Mean peri-tumoral distance (± SD)** |
| --- | --- | --- | --- | --- |
| Breast cancer | 10 | 1.93 ± 1.03 mm | 3.29 mm | 1.86 ± 1.02 mm |
| Lung adenocarcinoma | 9 | 4.16 ± 1.86 mm | 5.88 mm | 3.64 ± 1.86 mm |
| Melanoma | 12 | 1.81 ± 0.99 mm | 3.37 mm | 1.66 ± 0.95 mm |
| Glioblastoma | 10 | 3.51 ± 1.21 mm | 5.26 mm | 1.31 ± 0.71 mm |

**Table S2. Biopsy characteristics according to tumor histology.** Mean peri-tumoral distances in lung adenocarcinoma biopsies were significantly greater than breast cancer (One-way ANOVA post-hoc Tukey, p<0.01), melanoma and glioblastoma samples (One-way ANOVA post-hoc Tukey, p<0.001).
